# Assessment of genetic diversity and volatile content of commercially grown banana (*Musa* spp.) cultivars

**DOI:** 10.1038/s41598-022-11992-1

**Published:** 2022-05-13

**Authors:** Vidya R. Hinge, Irfan M. Shaikh, Rahul L. Chavhan, Abhijit S. Deshmukh, Rahul Mahadev Shelake, Sandip A. Ghuge, Amol M. Dethe, Penna Suprasanna, Ulhas Sopanrao Kadam

**Affiliations:** 1 Department of Plant Biotechnology, Vilasrao Deshmukh College of Agricultural Biotechnology (Vasantrao Naik Marathwada Agricultural University, Parbhani), Latur, Maharashtra India; 2 Division of Applied Life Science, Plant Molecular Biology and Biotechnology Research Center, Gyeongsang National University, Jinju, Gyeongnam 52828 Republic of Korea; 3 Agricultural Research Organization (ARO), The Volcani Institute, P. O. Box 15159, 7505101 Rishon LeZion, Israel; 4 Homi Bhabha National Institute (HBNI) and Nuclear Agriculture and Biotechnology Division, Bhabha Atomic Research Center, Mumbai, India; 5 Present Address: Division of Applied Life Science, Plant Molecular Biology and Biotechnology Research Center, Gyeongsang National University, Jinju, Gyeongnam 52828 Republic of Korea

**Keywords:** Genetic techniques, Mass spectrometry, Metabolomics, Plant biotechnology, Natural variation in plants, Plant biotechnology, Plant breeding, Plant molecular biology

## Abstract

Banana is an important fruit crop in the tropics and subtropics; however, limited information on biomarkers and signature volatiles is available for selecting commercial cultivars. Clonal fidelity is a major contributor to banana yield and aroma; however, there are no useful biomarkers available to validate clonal fidelity. In this study, we performed the molecular profiling of 20 banana cultivars consisting of diploid (AA or AB) and triploid (AAA or AAB or ABB) genomic groups. We screened 200 molecular markers, of which 34 markers (11 RAPD, 11 ISSR, and 12 SSR) yielded unequivocally scorable biomarker profiles. About 75, 69, and 24 allelic loci per marker were detected for RAPD, ISSR, and SSR markers, respectively. The statistical analysis of molecular variance (AMOVA) exhibited a high genetic difference of 77% with a significant FST value of 0.23 (*p* < 0.001). Interestingly, the UBC-858 and SSR CNMPF-13 markers were unique to Grand Nain and Ardhapuri cultivars, respectively, which could be used for clonal fidelity analysis. Furthermore, the analysis of banana fruit volatilome using headspace solid-phase microextraction-gas chromatography-tandem mass spectrometry (HS-SPME-GCMS) revealed a total of fifty-four volatile compounds in nine banana cultivars with 56% of the total volatile compounds belonging to the ester group as the significant contributor of aroma. The study assumes significance with informative biomarkers and signature volatiles which could be helpful in breeding and for the authentic identification of commercial banana cultivars.

## Introduction

Banana (*Musa* spp.) is one of the world’s major fruit crops belonging to the *Musaceae* family. It is native to the tropical region of Southeast Asia^1^. India ranks first in banana production, with 31.5 million tons accounting for 26.43% of global output^2^. Banana is rich in carbohydrates, vitamin B, and a good source of sodium, potassium, calcium, and magnesium^3,4^. It is a perennial with a faster relative growth rate than other fruit crops and produces fruits throughout the year. Banana cultivars are propagated asexually via micropropagation or suckers^5^.

The cultivated edible *Musa* spp. originated from two wild species, *Musa acuminata* Colla (A genome) and *Musa balbisiana* Colla (B genome); both belong to the section *Eumusa.* The banana cultivars possess good genetic diversity with genome groups of AA, AB, AAA, AAB, ABB, AABB, AAAB, or ABBB type resulting from inter or intra-specific hybridization of *M. acuminata* and *M. balbisiana*^1,6^*.* Morphological data have suggested that *Musa* spp. is diverse and showed some well-defined unifying characteristics, indicating the genomic constitution. Complex genome structure and phylogenetic relationship among the hybrid cultivars and wild types need further exploration. Due to its narrow genetic base, banana cultivation is prone to pests and diseases^7^. Further, this is exacerbated by devastating extremities of abiotic stresses induced by climate change and global warming^8^. Identifying genotypes with high potential is critical for an effective, economical, and environmentally friendly approach to boosting banana productivity. The wild germplasm (BB-rich genomes) possesses hardiness and could act as potential donors in developing high-performing cultivars through molecular breeding^9,10^. In this regard, the utility of molecular tools in selecting and developing biotic and abiotic stress-resistant genotypes is of paramount importance in banana breeding programs^8,11,12^.

Researchers and farmers essentially require authentic high-yielding cultivars to maximize the output. However, traditional methods based on the phenotypic selection are complex and irreproducible to identify authentic cultivars. Hence, it is necessary to search for and develop novel molecular biomarkers which are stably inherited and unperturbed by environmental deviations^11^. Such molecular markers can be valuable for assessing genetic diversity to complement traditional breeding methods. Moreover, the genetic content (molecular identity information) is highly stable and not influenced by environmental factors; hence differences among genotypes could be discerned at the DNA level. Molecular markers are being widely used in plants to detect the genetic basis of variation, genetic relationships within germplasm, identification of duplicate accessions, and genetic fidelity testing. Several marker-assisted traits have been used in banana breeding^13^. Restriction fragment length polymorphism (RFLP)^1,7,14^; random amplified polymorphic DNA (RAPD)^9^ amplified fragment length polymorphism (AFLP)^15–17^; and microsatellites or simple sequence repeats (SSRs) and inter-simple sequence repeats (ISSRs)^18^ have been employed in the characterization and evaluation of genetic diversity in *Musa* species. Amongst these markers, SSRs show a higher degree of polymorphism and codominant inheritance mode and are reproducible^11,19^.

Fruit volatile (the total volatile compounds in banana fruit) is the vital contributor to banana appeal and aroma characteristics which influence fruit delicacy, quality, and consumer preference. Banana fruits are rich in flavor and aroma with the occurrence of more than 250 volatile compounds of several chemical classes, including esters, ketones, terpenes, and aldehydes^20^. Despite the limited literature on banana fruit volatiles and their contribution to aroma^21,22^, the role of banana volatiles has been indicated in the resistance mechanism to some important fungal diseases like Sigatoka^23^ and insect pests. The study of volatile components from banana fruits is also very important to assess the effect of changing climatic conditions on fruit quality^*^^1^. In light of these, we sought to understand the characteristic volatilome of each banana cultivar. Profiling of volatile compounds representing characteristic aroma and flavor of banana germplasm by using headspace solid-phase microextraction (HS-SPME) followed by gas chromatography and mass spectrometry (GC–MS)^24,25^ provides an additional tool for characterization of banana germplasm which could be utilized for decoding the flavor of diverse banana cultivars and breeding for enhanced sensory quality^20,22,25,26^.

In the present study, we have used various molecular markers to evaluate the genetic diversity among commercially grown banana cultivars belonging to different genomic groups. This study revealed the identification of cultivars-specific biomarkers which may help to establish genetic relationships. Additionally, the HS-SPME and GC–MS were used to develop the volatile profile of the genotypes. Such markers will enable plant breeders to select banana cultivars with better aroma, flavor, and desirable characteristics, besides helping researchers and farmers verify commercially grown varieties’ genetic fidelity.

## Materials and methods

### Plant genetic resources

The leaf samples of twenty different germplasm of banana comprising diploid and triploid genomes were collected from the Banana Research Station, Nanded affiliated to Vasantrao Naik Marathwada Krishi Vidyapeeth (VNMKV), Parbhani (Table 1). The study complies with relevant institutional biosafety regulations and guidelines. The genomic DNA was extracted using the modified CTAB method^27^ followed by RNase treatment. The qualitative and quantitative analysis of genomic DNA was done using a spectrophotometer and by resolving on 0.8% agarose gel. The Genomic DNA was diluted to 50 ng/µL and subsequently used for RAPD, ISSR, and SSR fingerprint analysis.

**Table 1:** Banana genotypes used in the present study.

Sr. no	Genotypes	Genome	Ploidy	Characteristics
1	Namarai	AA	2X	Resistance to banana *pseudostem* weevil
2	Ankur	ABB	3X	Drought tolerance and female fertile
3	SafedVelchi	AB	2X	Small, firm-fleshed and sweet fruits, Plants grown as intercrop in coconut and arecanutgardens
4	Bhurkel	ABB	3X	–
5	Bluggoe	ABB	3X	Cooking type, Fruits are starchy in texture and resistant to Panama disease
6	Borkal Baista	ABB	3X	Non-seeded, Culinary and dessert purposes
7	Karpuravalli	ABB	3X	Drought tolerant and female fertile
8	Birbutia	ABB	3X	Moderately tolerant to drought,male and female fertile
9	Karimbontha	ABB	3X	–
10	Karpura Chakrakeli	AAB	3X	Table banana, medium sized fruit with distinct tip, Resistant to Panama disease, Leaf Spot, tolerant to poor soils and drought
11	Rajeli	AAB	3X	Desert type, used to prepare fried chips called Sukeli
12	Ardhapuri	AAA	3X	Dessert type banana cultivar
13	Grand Nain	AAA	3X	Highly susceptible to water stress, Female sterile
14	NRCB-3	AAAB	4X	Cooking type, cold tolerant, dwarf banana
15	Elavazhai	BB	2X	Wild type cultivar, Less tolerant to drought,male and female fertile Male and female fertile
16	Lamby	ABB	3X	Moderately drought tolerant, male fertile,female sterile
17	Ney Poovan	AB	2X	Fruits are slender and less vigorous. Drought tolerant, Reduced female fertile, Resistant to *Fusarium* and leaf spot, susceptible to banana bract mosaic virus
18	Nendran	AAB	3X	For Chips making, Highly susceptible to Banana Bract Mosaic Virus (BBMV), *pseudostem* borer, rhizome weevil and nematodes. Resistant to Panama wilt and leaf spot diseases
19	Rasthali	AAB	3X	Table fruit, Fruits with white flesh, firm, tasty with a characteristic flavor, highly resistant to Leaf Spot but very susceptible to 'Panama' disease
20	Neyvazhai	AAB	3X	–

### RAPD, ISSR, and SSR genotyping

A total of 200 primers were used for pre-screening and of which 11 RAPD, 11 ISSR, and 12 SSR primers were used for genotyping twenty banana cultivars (Table 2). Banana cultivars representing each genome type i.e. Namarai from diploid, Birbutia and Grand Nain from triploid, and NRCB-3 from tetraploid were selected for pre-screening. Among the 75 RAPD, 60 ISSR and 65 SSR primers; the primers showing amplification in the all above selected banana cultivars were shortlisted. PCR reactions for RAPD genotyping were carried out in 25.0 µL reaction volume containing 2.5 µL 10× PCR assay buffer with MgCl_2_(10×), 0.5 µL dNTP mix (10 mM), 0.33 µL *Taq* DNA polymerase (3 U/µL), 2 µL Primer (10 µm/µL) and 1 µL template DNA (50 ng/µL). ISSR PCR reaction of 25.0 µL comprised 2.5 µL 10× PCR assay buffer with MgCl_2_, 0.5 µL dNTP mix (10 mM), 0.4 µL *Taq* DNA polymerase (3 U/µL), 1 µL Primer (10 µm/µL) and 1 µL template DNA (50 ng/µL). The SSR genotyping were performed in 25.0 µL reaction with 2.5 µL 10× PCR assay buffer, 1.5 µL MgCl_2_(25 mM)_,_ 0.5 µL dNTP mix (10 mM), 0.5 µL *Taq* DNA polymerase (3 U/µL), 1.5 µL each forward and reverse primer (10 µm/µL) and 1.5 µL template DNA (50 ng/µL). The RAPD PCR was carried out in Eppendorf thermal cycler for 5 min at 94 °C, followed by 40 cycles of 45 s at 94 °C, 1 min at 36 °C, 1 min at 72 °C with a final extension time of 10 min at 72 °C. The thermal cycler conditions for ISSR genotyping were initial denaturation at 94 °C for 5 min followed by 40 cycles of 45 s at 94 °C, 1 min at 52 °C, 1 min at 72 °C with a final extension time of 10 min at 72 °C. A thermal cycler for SSR PCR was carried out at 94 °C for 4 min, 35 cycles of 94 °C for 40 s, 54 °C for 30 s, and 72 °C for 40 s followed by a final extension of 10 min at 72 °C. The RAPD and ISSR PCR amplified products were resolved on 1.5% agarose gel at 100 V. Whereas, SSR PCR products were resolved on 2.5% agarose gel at 100 V, followed by ethidium bromide staining. The images were captured by a gel documentation system (AlphaImager, TM-2200).

**Table 2:** RAPD, ISSR and SSR primers used for genotyping twenty banana cultivars.

Sr. no	RAPD primers Name (Seq 5′–3′)	ISSR primers Name (Seq 5′–3′)	SSR primers Name (Seq 5′–3′)	
1	**OPC-08** TGGACCGGTG	**UBC-810** (GA)8T	**AGMI93/94**	F(AACAACTAGGATGGTAATGTGTGGAA)
				R(GATCTGAGGATGGTTCTGTTGGAGTG)
2	**OPA-10** GTGATCGCAG	**UBC-820** (GT)8C	**Ma 1/16**	F (TTTGCCTGGTTGGGCTGA)
				R (CCCCCCTTTCCTCTTTTGC)
3	**OPA-11** CAATCG CCGT	**UBC-826**(AC)8C)	**Ma 3/2**	F(GGAACAGGTGATCAAAGTGTGA)
				R(TTGATCATGTGCCGCTACTG)
4	**OPA-12** TCGGCG CCGT	**UBC-858**(TG)8RT	**Ma 3/103**	F(TCGCCTCTCTTTAGCTCTG)
				R(TGTTGGAGGATCTGAGATTG)
5	**OPA-13** CAGCACCCAC	**ISSR-825** (AC)8T	**CNPMF-4**	F(TCTGTACGTCTGCCTTGCAC)
				R(TGCAGGAGGTGGATCCATAG)
6	**OPA-17** GACCGCTTGT	**ISSR-841** (GA)8YC	CNPMF-8	F(ATCGAGGAATTTGGGAGAGG)
				R(ATCCACAATCCGATCAGCTC)
7	**OPA-18** AGGTGACCGT	**ISSR-857** (AC)8YG	**CNPMF-9**	F(CCTTCATCATCATCACGGC)
				R(ACCACGACCTCCTCCTCTTC)
8	**OPD-15** CATCCGTGCT	**ISSR-815** (CT)8G	**CNPMF-10**	F(CACATCACACGCTCTGCTTC)
				R(TTTTTCGGCTGATCCAATTC)
9	**OPD-18** GAGAGCCAAC	**ISSR-818** (CA)8G	**CNPMF-12**	F(CAAAGTTTGAAAGGGAGGGG)
				R(CTCGGACCACTAGCTTCCTG)
10	**OPD-20** ACCCGGTCAC	**ISSR-822** (TC)8A	**CNPMF-13**	F(GGGATGGCGCACTTCTTC)
				R(AATCCGGGTTGTAAGGAACC)
11	**OPN-02** ACCAGGGGCA	**ISSR-827** (AC)8G	**CNPMF-16**	F(TGTGTGACTACTCCCGGTTTC)
				R(GTCTGCTGCTCTATCCCGAG)
12	–	–	**CNPMF-19**	F(GTGTTCGAGAGCTTTCAGCC)
				R(AGAACAATCAAGCCAGCAGC)

Significant values are in [bold].

### Data scoring and analysis

All visible and unambiguously scorable fragments amplified by the RAPD, ISSR, and SSR primers were scored as scorable fragments. Amplification profiles of the germplasm were compared, and bands of DNA fragments were scored as present (1) or absent (0). The DNA fingerprint data of the primers were utilized to estimate genetic similarity based on a number of shared amplification products^28^. The equation used was the number of shared amplification products = 2 × (number of typical bands between any two lanes)/(Total number of bands in the same two lanes). Genetic relationship among the germplasm was estimated with the dendrogram constructed through an unweighted pair group of arithmetic means using (UPGMA)^29^ and NTSYS pc 2.02 software. Further, the population genetic structure was analyzed based on SSR amplicons score as allele size (bp) using the Bayesian clustering method and STRUCTURE v.v 2.3.3 software^30^. The admixture model and correlated allele frequencies were applied for the estimation of ancestry fractions of each cluster. The value of K (range 1–10) was estimated using five independent runs and a burn-in period of 20,000 followed by 200,000 MCMC (Markov Chain Monte Carlo) repetitions. The web-based software STRUCTURE HARVESTER version 0.6.92 was used to determine the optimum K value using the log probability of data, LnP(D) based on the rate of change in LnP(D) between successive K^31^.

### Volatile profiling of Banana cultivars using HS-SPME GCMS

The unripe banana fruits of available banana cultivars were collected from the Banana Research Station, Nanded, and kept for ripening at 20 °C.The pulp of fully ripe banana fruits was used for volatile analysis. A blended pulp juice from the three banana fruits of each banana cultivar was prepared separately, and 16 g of juice in a 30 ml glass vial tightly sealed with a Teflon septum with a plastic cap was used for volatile analysis. Optimized SPME parameters, i.e., sample equilibration for 15 min, adsorption for 60 min at 25 °C, and desorption time of 10 min, were used for GCMS sampling^32^. The 430 GC analyzed the volatile compounds, and 210 MS (Varian) gas chromatograph-mass spectrometer (GC–MS) equipped VF-5 MS column with helium as the carrier gas at a flow rate of 1.0 mL/min was used for detection of m/z ratio of fragmented ions and derived spectrum. The injection port was lined with a 0.75 mm, i.e., splitless glass inserter, and maintained at 200 °C. The oven temperature was programmed to rise from 50 to150 °C at 2 °C/min and the total GC run time was 55 min. MS transfer line was maintained at 290 °C, ionization energy was 70 eV, and the mass range was 50–550 m/z. The volatile compounds were identified by comparing the results obtained with the reference mass spectra from the NIST library (NIST98, version 2.0, Gaithersburg, USA) and retention index values obtained using a standard mixture of alkanes (C8–C20).

## Results

Various molecular markers have been used extensively to screen the diversity and develop biomarkers for clonal identity in crops, including banana^9,17,33^. Here we employed three different markers—RAPD, ISSR, and SSR to search the cultivar-specific biomarkers. The molecular marker provided genetic information and assisted in finding out biomarkers specific to Grand Nain and Ardhapuri cultivars. Additionally, we performed the volatile profiling of banana cultivars to characterize the compounds that contribute to the characteristic aroma of the banana fruits.

### Screening of diploid and triploid cultivars of banana (*Musa* spp) predominantly cultivated in India

RAPD profiling of twenty banana cultivars produced polymorphic DNA fingerprint patterns and generated 725 polymorphic amplicons. Most RAPD primers produced 100% polymorphism (except OPA-13, OPA-18, OPD-15, and OPN-02, with an average polymorphism of 89.97%; Tables 3, 4, 5). Additionally, we recorded the average polymorphic bands per genotype ranging from 4.95 to 1.75 with a median value of 3.29. Moreover, the average number of loci per primer ranged from 5 to 10, with a median score of 6.81 loci per primer. The RAPD primer OPD-18 amplified the highest allelic loci, i.e., 10 followed by primers OPA-13, OPD-20, and OPN-02, each with 08 allelic loci. We evaluated the allele size of the RAPD profile, and it ranged from 0.1 to 1.5 kb. The polymorphic information content (PIC) value ranged from 0.43 for OPA-17 and OPA-10 to 0.25 for OPA-11 (Table 3). Furthermore, the average Shannon’s diversity index (H), expected heterozygosity (He), resolution power (RP), and marker index (MI) values for RAPD primers were 0.53, 0.35, 7.11, and 2.62, respectively. The RAPD primers OPD-18 and OPA-13 have recorded the highest resolution power and MI value. Overall, the RAPD marker-assisted molecular profiling produced highly polymorphic content.

**Table 3:** RAPD polymorphism.

Sr. no	Primer name	Total polymorphic amplicons/Total amplicon	Polymorphism %	Average polymorphic band/Germplasm	Total loci	PIC	Na	Ne	I	He	uHe	RP	MI
1	OPC-08	61/61	100.00	3.05	6.0	0.40	2.000	1.414	0.412	0.261	0.267	5.50	2.8
2	OPA-10	66/66	100.00	3.30	7.0	0.43	2.000	1.583	0.538	0.356	0.366	6.10	3.01
3	OPA-11	57/57	100.00	2.85	5.0	0.25	2.000	1.781	0.620	0.431	0.442	7.20	1.25
4	OPA-12	61/61	100.00	3.05	6.0	0.36	2.000	1.698	0.569	0.389	0.399	5.30	2.16
5	OPA-13	94/114	82.46	4.70	8.0	0.33	1.889	1.643	0.536	0.368	0.378	10.9	2.64
6	OPA-17	64/64	100.00	3.20	7.0	0.43	2.000	1.486	0.472	0.305	0.312	7.2	4.3
7	OPA-18	38/58	65.52	1.90	5.0	0.36	2.000	1.662	0.559	0.378	0.388	5.8	2.16
8	OPD-15	35/55	63.64	1.75	5.0	0.31	2.000	1.599	0.538	0.358	0.368	5.3	1.24
9	OPD-20	79/79	100.00	3.95	8.0	0.36	2.000	1.553	0.502	0.330	0.339	6.5	2.52
10	OPD-18	99/99	100.00	4.95	10	0.37	2.000	1.675	0.535	0.367	0.377	11.8	4.07
11	OPN-02	71/91	78.02	3.55	8.0	0.34	2.000	1.562	0.535	0.353	0.362	6.6	2.72
Mean		65.91/73.18	89.97	3.29	6.81	0.36	1.99	1.61	0.53	0.35	0.36	7.11	2.62

**Table 4:** ISSR polymorphism.

Primer name		Total polymorphic amplicons/Total amplicon	Polymorphism %	Average polymorphic band/Germplasm	Total loci	PIC	Na	Ne	I	He	uHe	RP	MI
1	ISSR-815	81/101	80.10	4.05	8	0.35	2.000	1.469	0.420	0.275	0.282	5.7	2.8
2	ISSR-818	94/94	100.00	4.70	8	0.42	2.000	1.417	0.418	0.265	0.272	5	3.36
3	ISSR-822	57/97	58.76	2.85	7	0.22	2.000	1.516	0.467	0.306	0.314	7.3	1.76
4	ISSR-827	63/83	75.90	3.15	7	0.37	2.000	1.486	0.482	0.310	0.318	8.1	2.96
5	ISSR-825	19/39	48.72	0.95	5	0.26	1.600	1.178	0.249	0.140	0.144	1.5	1.04
6	ISSR-841	40/80	50.00	2.00	6	0.33	2.000	1.544	0.481	0.317	0.326	8.9	3.3
7	ISSR-857	71/71	100.00	3.55	6	0.41	2.000	1.746	0.597	0.413	0.423	7.6	4.1
8	UBC-810	112/112	100.00	5.60	9	0.35	2.000	1.602	0.524	0.350	0.359	10.9	3.5
9	UBC-820	33/53	62.26	1.65	5	0.31	1.750	1.448	0.414	0.275	0.282	4.6	0.93
10	UBC-826	68/68	100.00	3.40	5	0.28	1.500	1.172	0.207	0.125	0.129	5.1	0.84
11	UBC-858	38/38	100.00	1.90	3	0.12	2.000	1.609	0.562	0.376	0.385	5.6	0.24
Mean		61.45/76	79.62	3.07	6.27	0.31	1.90	1.47	0.44	0.29	0.29	6.39	2.26

**Table 5:** SSR polymorphism.

Primer name		Total Polymorphic amplicons/Total amplicon	Polymorphism %	Average polymorphic band/Germplasm	Total loci	PIC	Na	Ne	I	He	uHe	RP	MI
1	CNMPF-8	22/22	100.00	1.1	3	0.12	2.000	1.212	0.256	0.149	0.152	2.1	0.36
2	CNMPF-9	30/30	100.00	1.2	3	0.00	2.000	1.526	0.477	0.315	0.323	3.0	0.00
3	CNMPF-10	18/18	100.00	1.0	2	0.32	2.000	1.573	0.481	0.319	0.327	1.9	0.64
4	CNMPF-12	20/20	100.00	1.0	1	0.00	2.000	1.532	0.531	0.347	0.356	1.9	0.0
5	CNMPF-13	23/43	53.49	1.15	4	0.26	2.000	1.507	0.475	0.310	0.318	4.1	1.04
6	CNMPF-16	19/19	100.00	0.95	1	0.09	2.000	1.532	0.531	0.347	0.356	1.9	0.09
7	CNMPF-19	34/54	62.10	1.7	5	0.34	2.000	1.579	0.530	0.350	0.359	5.1	1.7
8	CNMPF-21	17/17	100.00	0.85	1	0.26	2.000	1.903	0.668	0.475	0.487	1.7	0.26
9	Ma-1/16	30/30	100.00	1.5	2	0.37	2.000	1.718	0.599	0.411	0.421	3.6	0.74
10	Ma-3/2	19/19	100.00	0.95	1	0.09	2.000	1.532	0.531	0.347	0.356	1.9	0.09
11	Ma-3/103	20/20	100.00	1.0	2	0.09	2.000	1.532	0.531	0.347	0.356	1.9	0.18
12	Agmi 93/94	17/37	45.95	0.85	2	0.29	2.000	1.755	0.609	0.421	0.432	3.5	0.58
Mean		18.08/26.42	71.87	0.9	2	0.19	2	1.58	0.52	0.34	0.35	2.72	0.47

Na: no. of different alleles, Ne = NO. OF EFFECTIVE ALLeles = 1/(p^2 + q^2), I = Shannon's information index =  − 1* (p * Ln (p) + q * Ln(q)), He: expected heterozygosity = 2 * p * q, uHe = unbiased expected heterozygosity = (2N/(2N–1)), RP = resolution power, MI = marker index.

Next, the ISSR primers were used to evaluate the molecular diversity of banana cultivars. The ISSR markers showed an average of 79.62% polymorphism. Among all ISSR markers used, five ISSR primers—ISSR-810, ISSR-818, ISSR-826, ISSR-857, and ISSR-858 generated 100% polymorphism (Table 4). The number of loci observed per primer varied from 3 to 9, with an average of 6.27 loci per primer. The PIC value for ISSR primer ranged from 0.12 to 0.42, with an average of 0.31. The parameters such as H, He, RP, and MI values measured using ISSR primers were: 0.44, 0.29, 6.39, and 2.26, respectively (Table 5). The ISSR primers UBC-810, ISSR-841, and ISSR-827 were found to provide significant molecular markers with the highest resolution power and marker index value. Hence, the ISSR yielded a highly informative marker profile that successfully profiled selected genotypes of *Musa* spp.

Additionally, SSR markers were also used to gain insights into selected genotypes of banana cultivars. The SSR marker produced polymorphic bands ranging from 1 to 5 loci per primer, and a total of 217 unique amplicons were recorded (Table 5). The SSR marker analysis yielded an average polymorphism of 71.86%. The indexes like H, He, RP, and MI values calculated using SSR primers were 0.19, 0.52, 0.34, 2.72, and 0.47, respectively. Seven locus-specific SSR markers, Ma3/103, CNMPF-8, CNMPF-10, CNMPF-21, CNMPF-16, Ma1/16, and Ma3/2, showed a 100% polymorphic pattern. The highest PIC value of 0.37 was reported by the SSR primer Ma1/16. The SSR primers CNMPF-13, CNMPF-19, and Ma1/16 were significantly powerful in characterizing banana genotypes with the highest RP and MI value.

### Identification and assignment of cultivar-specific markers for clonal fidelity analysis

Molecular screening and data analysis provided several polymorphic markers for diploid and triploid genotypes of the banana crop. This study identified several cultivar-specific biomarkers using RAPD, ISSR, and microsatellite markers (Table 6, Fig. S1). We further analyzed the molecular data and assigned the biomarkers specific to the cultivar for clonal fidelity analysis in commercial agriculture. The RAPD primer OPC-08 has shown a particular fragment of 750 bp to Safedvelchi and Elavazhai cultivars (Fig. S1A). At the same time, the RAPD primer OPD-18 has shown a 1.4 kb specific fragment in cultivar Grand Nain, Ardhapuri, and Rasthali (Fig. S1B).

**Table 6:** Identification of genome/cultivar specific biomarker in banana.

Sr. no	Biomarker specific	Banana cultivar	Biomarker annotation detail
**A. RAPD**			
1	OPC-08 _(~0.75 Kb)_	Safed Velchi and Elavazhai	
2	OPD-18 _(~ 1.4 kb)_	Grand Nain , Ardhapuri and Rasthali	
3	OPD-20_(~1.5 Kb)_	Neypoovan	
**B. ISSR**			
6	ISSR-818_(~0.9 kb)_	Grand Nain and Ardhapuri	Genic Marker: KU977463.1, AM950411.1, XR_001975600.1, AM950478.1
7	ISSR-827_(~250 bp)_	Ankur	Genic Marker: EF467424.1, AF414128.1, AM950411.1, XM_009414952.2
8	ISSR-827_(~ 0.7 Kb),_	Grand Nain	
9	ISSR-825_(~ 1 Kb)_	Lamby	Genic marker;XM_009421366.2, XM_018818029.1, KU977463.1,
10	UBC-858_(~ 0.4 Kb)_	Ankur	Microsatellite marker ; AM950338.1 (398 bp), AM950477.1(236 bp) Genic marker: Accesion number: MF429831.1, XM_009415807.2,XM_009420089.2 XM_009420482.2
11	UBC-858_(~ 0.2 Kb)_	Grand Nain	
**C. SSR**			
12	CNMPF-8_(~0.5 kb)_ CNMPF-9_(~0.9 kb)_	Ardhapuri	Genic marker: FM878688.1 XM_009407999.2, XM_009418853.2
13	CNMPF-8_(~0.2 kb)_	Safed Velchi	Genic marker: FM878688.1
14	CNMPF-13_(~0.3 kb)_	Ardhapuri	Genic marker: XM_009386686.2

Additionally, the RAPD profile of OPD-20 primer generated a unique band of 1.5 kb for the Neypoovan cultivar (Fig. S1C). The ISSR marker analysis of cultivars Grand Nain and Ardhapuri yielded a specific 0.9 kb fragment during fingerprinting using the ISSR-818 primer (Fig. S1D). The ISSR-818 primer was found to be located in the gene (KU977463.1) Musa acuminata AAA Group Constans-like 3b (COL3b) mRNA, complete CDS with E Value 0.062. Thus ISSR-818 was identified as a genome A specific genic biomarker. The cultivar Ankur yielded a 0.250 kb unique single fragment with an ISSR primer ISSR-827 and UBC-858 (Fig. S1E,G). The Grand Nain cultivar displayed a unique single specific amplicon of 0.7 kb with the primer ISSR-827 and a 0.2 kb amplicon for the primer UBC-858. The ISSR-827 marker yielded 250 bp and 700 bp unique alleles for the cultivars Ankur and Grand Nain, respectively. The banana cultivars Ankur with genome ABB representing *M. balbisiana* genetic background. Thus ISSR-827 microsatellite marker showed 100% similarity with *M. balbisiana* microsatellite Mbc08 sequence (0.281 kb; Acc. No. EF467424.1). The ISSR-827 was yielding 0.7 kb unique fragment in banana cultivar Grand Nain to confirm the presence *M. acuminata* calmodulin-like protein mRNA, partial CDS (765 bp) (AF414128.1) in *M. acuminata* Grand Nain (AAA) banana cultivar. Thus ISSR-827 yielding ⁓750 bp fragment was identified as a genic biomarker specific to the Grand Nain banana cultivar. The ISSR marker UBC-858 displaying 0.4 kb unique fragment in Ankur and 0.2 kb in Grand Nain is identified as a genic microsatellite biomarker as it was showing similarity with the *M. acuminata* subsp. *malaccensis* transcription factor bHLH112 (LOC103998874), transcript variant X2, mRNA, *M. acuminata* subsp. *malaccensis* abscisic acid 8'-hydroxylase 3-like (LOC103999378), transcript variant X1, mRNA, *M. acuminata* subsp. *malaccensis* indole-3-pyruvate monooxygenase YUCCA2-like (LOC103984381), mRNA etc., with E value 0.023 and confirmed its genic nature. Further, the ISSR-825 profile yielded a specific fragment of 1.0 kb (Fig. S1F) in the banana cultivar Lamby. The ISSR-825 represents the *M. acuminata* subsp. *malaccensis* pectinesterase 1-like (LOC103999580), mRNA (XM_009421366.2) (1370 bp), *M. acuminata* subsp. *malaccensis* uncharacterized LOC103999495 (LOC103999495), mRNA (1004 bp)( XM_018818029.1). The banana cultivar Ardhapuri produced a specific fragment of 0.5 kb in the DNA fingerprinting analysis using SSR primer CNMPF-8, and a 0.9 kb fragment was observed in the fingerprint of SSR CNMPF-9 (Fig. S1H,I). The CNMPF-8 and CNMPF-9 were identified as genetic markers showing similarity with *M. acuminata* subsp. *burmannicoides* var. Calcutta 4 mRNA containing microsatellite, clone MAC4-67K01-F, 595 bp linear mRNA (FM878688.1); and *M. acuminata* subsp. *malaccensis* uncharacterized LOC103989204 (LOC103989204), transcript variant X3, mRNA 925 bp linear mRNA (XM_009407999.2). The identified SSR and ISSR biomarkers were genic markers and represented transcripts of the Musa genome (Table 6). The detailed annotation of the SSR and ISSR biomarkers in the banana genome is listed in Table 6. This set of molecular markers could be further developed as the biomarker for the clonal fidelity testing of tissue culture-raised plantlets of cultivars of commercial significance.

### Estimation of genetic diversity using molecular clustering

**Figure 1:**
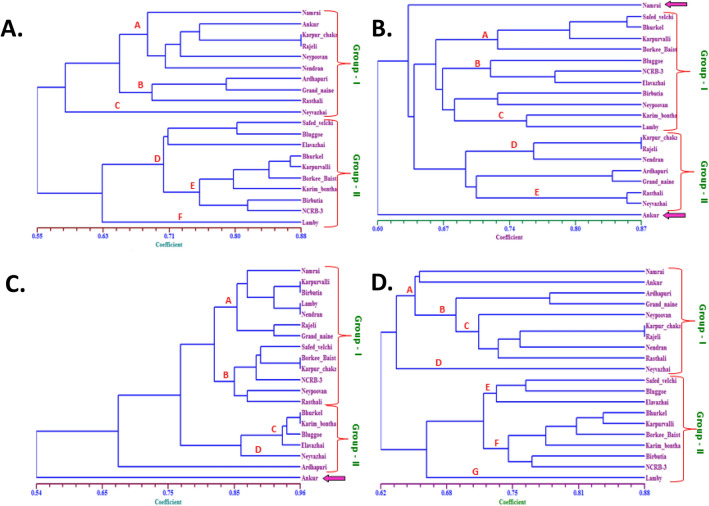
Dendrogram demonstrating relationship among twenty banana germplasm based on: (**A**) RAPD, (**B**) ISSR, (**C**) SSR, and (**D**) combined markers (RAPD, ISSR and SSR) analysis.

The genetic diversity of bananas has been carried out using different molecular markers, and significant success has been made for analyzing commercial cultivars and some wild germplasm^34,35^. However, not much is known about the local germplasm, which harbors important traits for adaptation to local climatic conditions and small-holdings. In this regard, genetic diversity analysis was carried out in the diverse banana germplasm. Molecular clustering analysis of 20 banana genotypes based on RAPD fingerprinting resulted in two significant subgroups comprising ten genotypes in each group with 55% similarity (Fig. 1A). Group-I comprised banana genotypes with AA, AB, AAB, ABB, and AAA genomes, including Namarai, Ankur, Karpura Chakrakeli, Rajeli, Ney Poovan, Nendran, Ardhapuri, Grand Nain, Rasthali, and Neyvazhai. Within Group-I, cultivars Karpura Chakrakeli and Rajeli were found to be closely related with 88% of similarity. Group-II consisted of nine banana cultivars carrying AB, BB, ABB, and AAB genomes, which consisted of Safed Velchi, Elavazhai, Bhurkel, Bluggoe, Karpuravalli, Birbutia, Karimbontha, NRCB-3, and Borkal Baista. The cultivars in Group-II were found to have at least 71% similarity level. Whereas the cultivar Lamby was observed to have 37% uniqueness from other cultivars in Group-II.

The phylogenetic analysis based on ISSR fingerprinting generated a dendrogram, which categorized 20 banana genotypes into two major groups in addition to the banana cultivars Namarai (AA) and Ankur (ABB) were clustered separately (Fig. 1A). Group I comprised banana cultivar of genome AB and BB type (Safed Velchi, Bhurkel, Karpuravalli, Borkal Baista, Bluggoe, NRCB-3, Elavazhai, Birbutia, Ney Poovan, Karimbontha and Lamby) with 70% of similarity level; while group II comprised of banana cultivars carrying AAA, AAB and ABB genomes, i.e., Karpura Chakrakeli, Rajeli, Nendran, Ardhapuri, Grand Nain, Rasthali, and Neyvazhai (Fig. 1B).

The clustering analysis helped to obtain a dendrogram based on the SSR fingerprinting data of twenty banana cultivars (Fig. 1C). The SSR analysis divided twenty banana cultivars into two major groups, cultivar like Ankur was grouped separately with 46% dissimilarity level. Group-I comprised of 13 banana cultivars, viz., Namarai, Karpuravalli, Birbutia, Nendran, Safed Velchi, Lamby, Rajeli, Karpura Chakrakeli, Grand Nain, Ney Poovan, Rasthali, NRCB-3, and Borkal Baista with 80% similarity; whereas group II comprised 6 cultivars, i.e., Bhurkel, Bluggoe, Karimbontha, Elavazhai, Neyvazhai, and Ardhapuri with 86% similarity index.

Consequently, we combined the RAPD, ISSR, and SSR fingerprinting data of twenty banana cultivars to construct a dendrogram (Fig. 1D). The combined molecular fingerprinting analysis revealed two major clusters, each comprised of 10 genotypes sharing 62% similarity. The first group contains Namarai, Ankur, Ardhapuri, Grand Nain, Ney Poovan, Karpura Chakrakeli, Rajeli, Neyvazhai, Nendran, and Rasthali. Among the genotypes in group I, Karpura Chakrakeli and Rajeli were genetically closer with 88% similarity, followed by genotypes Ardhapuri and Grand Nain with 81% similarity index. Whereas genotypes Neyvazhai, Namarai, and Ankur were grouped separately from other genotypes at a 47% dissimilarity level, Group-II comprised nine banana genotypes sharing 70% similarity. Lamby’s genotype was separated at a 35% dissimilarity level from other genotypes in group II. The dendrogram obtained after collective clustering described the precise grouping of all twenty banana genotypes than the individual marker-based grouping. The results confirmed the applicability of a three-way molecular marker system in genotyping with higher confidence in estimating cultivar diversity among banana diploid and triploid genotypes.

### Principle coordinate (PCA) and population structure analysis (PSA) revealed genetic distance among banana cultivars

**Figure 2:**
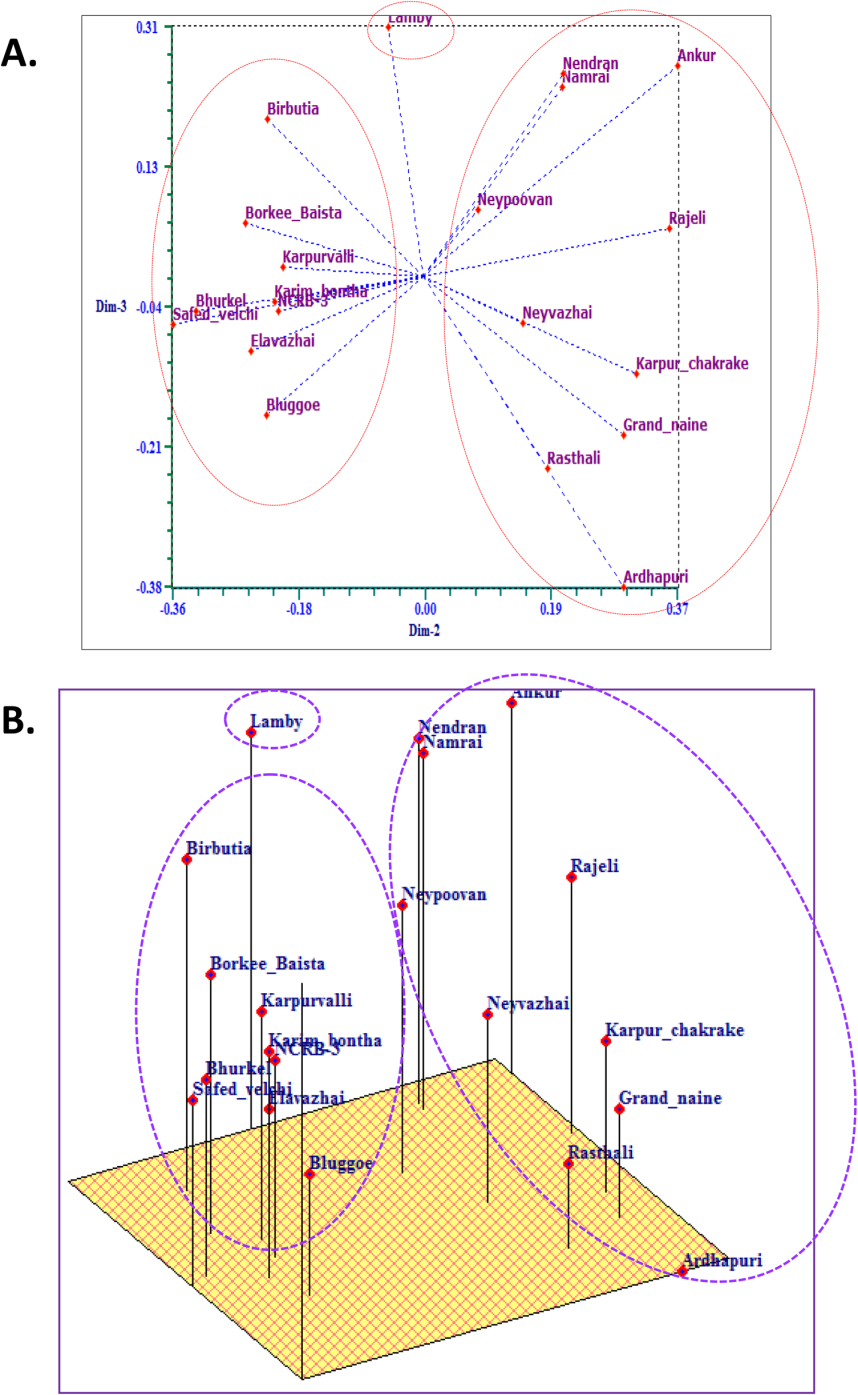
(**A**) 2-Dimensional principle coordinate analysis (PCA) analysis of RAPD, ISSR, and SSR combined marker data of banana Germplasm. (**B**) 3-Dimensional PCA of RAPD, ISSR, and SSR combined marker data of banana germplasm.

The two-and three-dimensional PCA based on genetic variance was performed, which helped to categorize banana germplasm into two distinct groups similar to the groups or clusters obtained during dendrogram analysis of the combined molecular marker system (Fig. 2A,B). PCA showed that the first three Eigenvalues explained 34% of the cumulative variation plotted to reveal diversity among banana germplasm. PCA corroborated cluster analysis results based on RAPD, ISSR, and SSR DNA fingerprinting. The cultivar Lamby was out-grouped and found genetically most distant.

**Figure 3:**
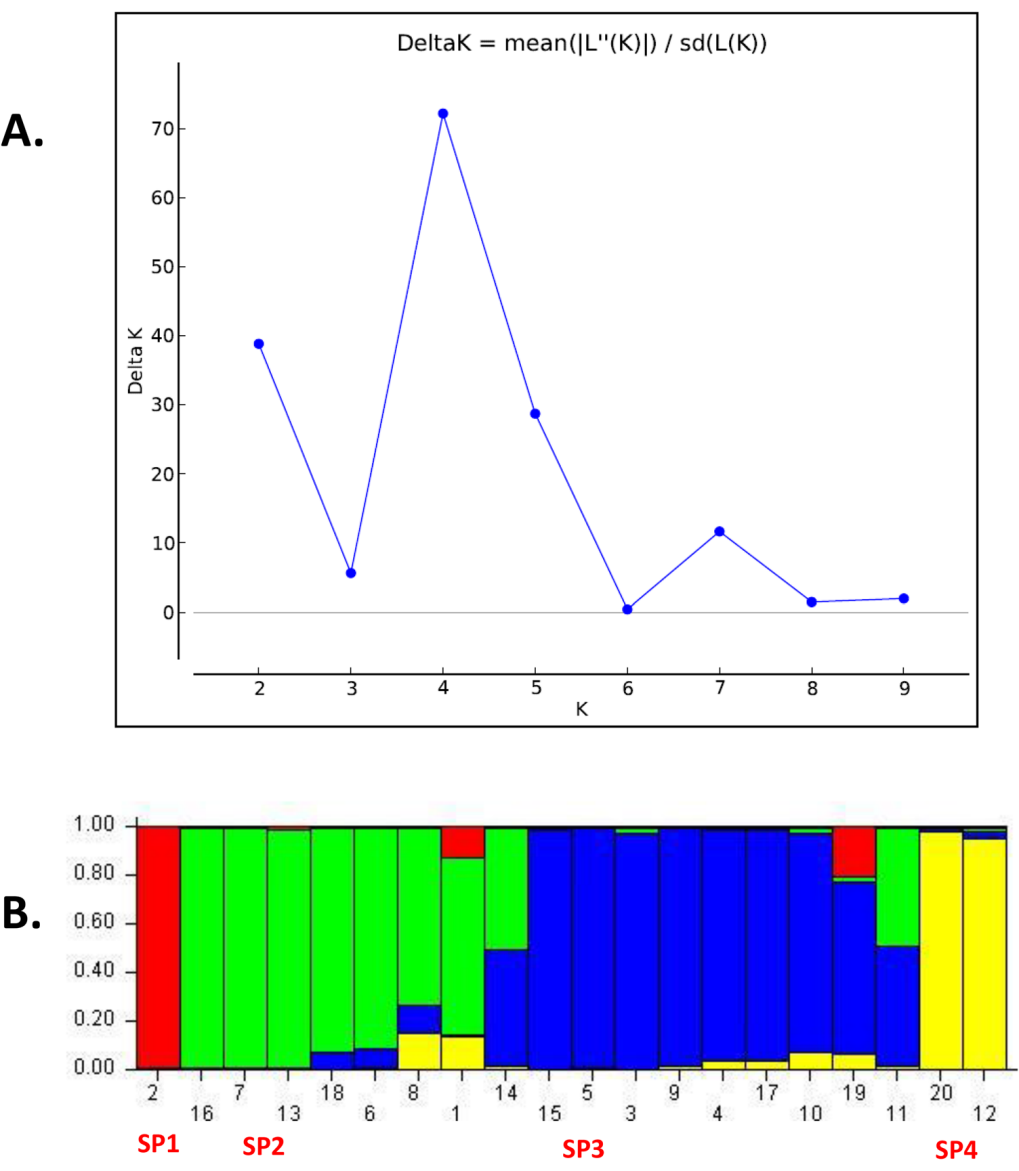
Population structure analysis depicting the genetic relationships among 20 banana genotypes based on SSR markers. (**A**) The structure analysis results based on a K-value. (**B**) Grouping of population into four subpopulation based on PSA.

The population structure of 20 banana genotypes was estimated using SSR data. The structure analysis results based on a K-value of 1–10 revealed a peak of dk at K = 4 (Fig. 3A), proposing four major populations (Fig. 3B). The probability of membership threshold (C = 0.44), ten banana genotypes were allocated to SP3 (as 7 pure and 3 admixture), seven genotypes into SP2 (as 5 pure and 3 admixture), two genotypes into SP4, and one genotype into SP1. The pairwise genetic distance among the population was estimated (see Tables 7, 8). The expected heterozygosity between members of the same population was observed as 0.1675 (SP1), 0.0674 (SP2), 0.1367 (SP3), and 0.1505 (SP4). The pairwise genetic differentiation among the four subpopulations was compared based on FST values; the pairwise FST values ranged from 0.171 to 0.555. A high genetic differentiation was observed between SP3 and SP4 with FST value of 0.171. The moderate differentiation was observed between other sub-populations i.e., FST = 0.172 for SP2 and SP3, FST = 0.171 for SP3 and SP4, FST = 0.2342 for SP2 and SP4, FST value 0.3748 for SP2 and SP1, and FST value 0.4987 for SP1 and SP4 (Table 8). Additionally, we performed AMOVA for all 20 banana genotypes considering four sub-populations to support this evidence. A highly significant FST value of 0.23 (*p* > 0.001) was obtained, suggesting the presence of overall high genetic differentiation among and within populations. Further, it was observed that 23% of genetic variation separated the four sub-populations, and 77% of the total variation existed within the sub-populations (Table 9).

**Table 7:** Estimation of gene diversity and heterozygosity in population based on SSR analysis.

Population	Total collection			
	SP1	SP2	SP3	SP4
No	01	07	10	02
Gene diversity	0.7687	0.7916	0.6755	0.6595
Heterozygosity	0.1675	0.0674	0.1367	0.1505

**Table 8:** Pairwise genetic differentiations among the subpopulations of entire set Populations.

Population	Total collection			
	SP1	SP2	SP3	SP4
SP1	0.0000	0.3784	0.5552	0.4978
SP2		0.0000	0.1721	0.2342
SP3			0.0000	0.1713
SP4				0.0000

**Table 9:** Analysis of molecular variance (AMOVA) of SSR marker for banana cultivars.

Source	Degrees of freedom (df)	Sum of squares (SS)	Mean squares (MS)	Estimated variance	Percentage of variation (%)	Stat	Value	P(rand Cdata)
Among population	03	39.752	13.251	0.859	23	Fst	0.230	0.001
Within population	36	92.048	5.753	2.876	77			
Total	39	131.800		3.735	100			

### Volatile profiling of banana cultivars

**Figure 4:**
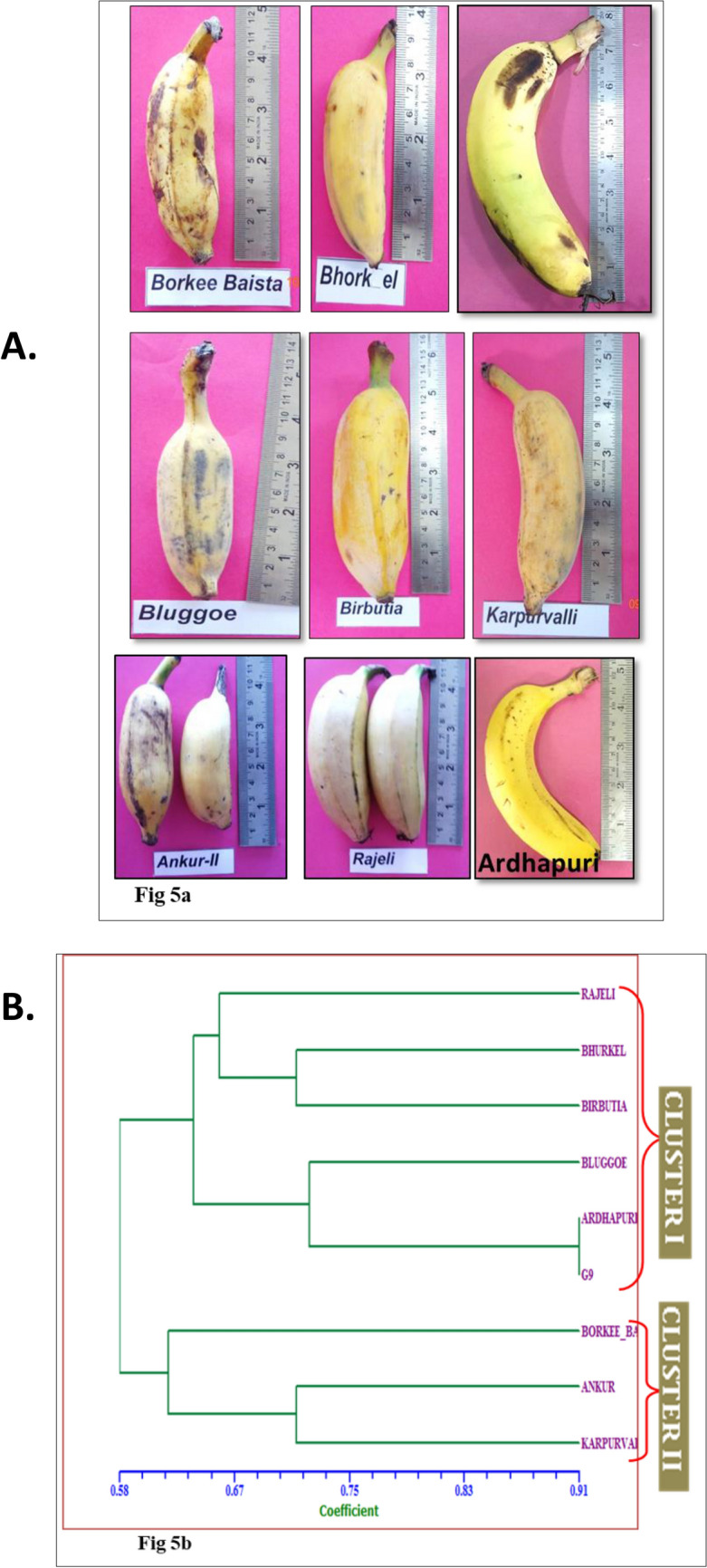
(**A**) Images showing the mature fruits of banana, some popular cultivars grown within India. (**B**) Dendrogram demonstrating relationship among nine banana genotypes based on the volatile compounds.

**Figure 5:**
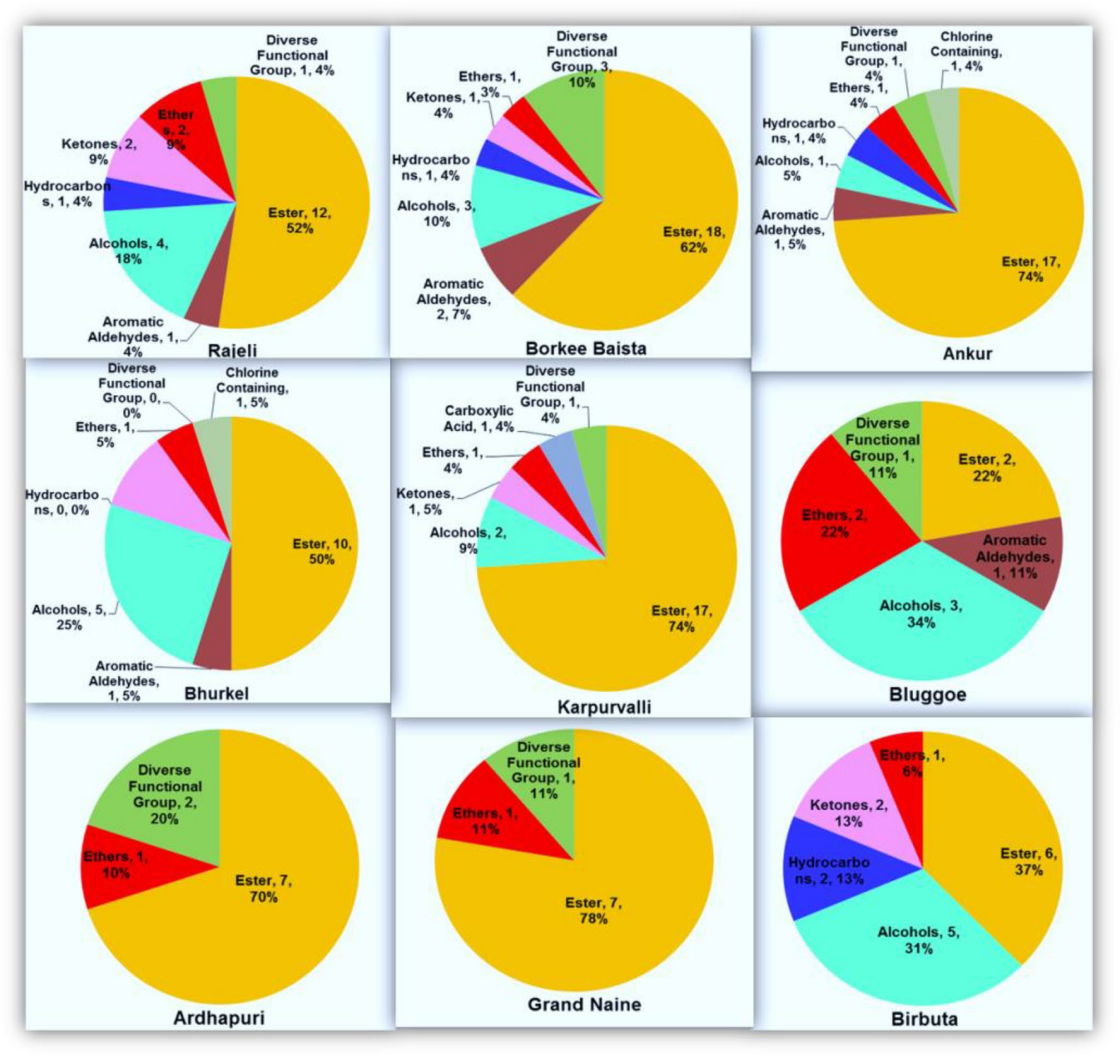
Volatile profile of selected nine banana cultivars.

Banana fruits are characterized by specific aroma profiles (volatilome), and considerable effort is needed to investigate aroma profiles during the ripening and post-ripening stages or storage of banana cultivars^26,36^. In the present study, we performed the volatile compounds profiling to understand the genotypic variation for volatilome of selected banana cultivars of the different genomic constitutions. The volatile compounds identified in banana fruits of nine different cultivars are depicted in Table 10. Maximum volatile compounds were identified in Borkal Baista i.e. 29, followed by 23 each in Rajeli, Ankur, and Karpuravalli, 20 in Bhurkel,16 in Birbutia, 10 in Ardhapuri, and 9 in Grand Nain. A total of fifty-four volatile compounds were identified from fruit samples of nine cultivars of banana and classified into nine unique chemical groups (Fig. 4, Table 10). Almost 56% of the total identified volatile compounds belonged to the ester group, while a minimum of about 2% belonged to carboxylic acid (Fig. 5). The ester compounds were predominantly observed in all the studied banana cultivars (Fig. 5). Among the nine banana cultivars, the highest number of volatile components, i.e., more than 20 i.e., approximately 37%, were recorded in fruits of the cultivars of Borkal Baista, Karpuravalli, Rajeli, Ankur, and Bhurkel. Therefore, these cultivars are suggested to be as rich in aroma and can be utilized for flavor enhancement of banana fruit by selective breeding. The highest number of ester compounds was recorded in Borkal Baista (18), followed by Ankur (17), Karapurvalli (17), Rajeli (12), Bhurkel (10), and Ardhapuri and Grand Nine (7 each) (Table 10). In commercially grown cultivars, Ardhapuri and Grand Nine ester compounds contributed 70–78% of the volatilome suggesting that ester compounds are a major source of banana flavor and aroma and enhance the aesthetic value of the fruit. Another factor that contributed to the aroma was the alcohol groups. We found the distribution of alcohols in Birbutia (5, 31%), Bhurkel (5, 25%), Bluggoe (3, 34%) Rajeli (4, 18%), and Borkal Baista (3, 10%). The alcoholic compounds are also an important component of flavor in the cultivars desired for wine production. Additionally, aldehyde compounds were observed in Bluggoe, Borkal Baista, Bhurkel, Rajeli, and Ankur. Aldehyde compounds have an essential role in aroma^37^. Moreover, the ketone compounds have an important role in the banana aroma. In the present study, ketones were detected in five banana cultivars (Rajeli, Bhurkel, Karpuravalli, and Birbutia), contributing to the flavor of these cultivars. Although several compounds such as hydrocarbons, carboxylic acid, chlorine-containing ethers, and diverse functional groups were detected in the volatiome analysis, previous studies suggested that these compounds do not contribute to banana aroma^26^.

**Table 10:** Volatile profile of banana cultivars.

Sr no	RT	Name of compound	RI		Rajeli	Borkal Baista	Ankur	Bhurkel	Karpuravalli	Bluggoe	Birbutia	Ardhapuri	Grand nain
			E	R									
**1. Ester**					12	18	17	10	17	2	6	7	7
1	4.624	Acetic acid, butyl ester	781	785	−	−	−	+	+	−	−	−	−
2	4.672	Butanoic acid, ethyl ester	782	785	−	+	−	−	−	−	−	−	−
3	6.557	1-Butanol,3-methyl-,acetate	865	855	−	+	+	+	+	−	−	−	−
4	6.931	n-Butyl ether	881	880.2	−	−	−	−	−	−	+	−	−
5	8.108	2-Buten-1-ol, 3-methyl-, acetate	916	905	+	−	−	−	−	−	−	−	−
6	9.617	Butanoic acid, 2-methylpropylester	949	953	+	+	+	−	+	−	−	+	−
7	11.637	Butanoic acid, butyl ester	994	995	+	+	+	+	+	−	−	−	−
8	12.146	Butanoic acid, 3-methyl-, 2-methylpropylester	1004	990	−	+	−	−	−	−	−	−	−
9	12.539	Acetic acid, hexyl ester	1011	1008	−	−	+	+	+	−	−	−	−
10	12.547	Propanoic acid, 2-methyl-, pentyl ester	1011	1019	+	+	−	−	−	−	−	−	−
11	12.86	4-Hexen-1-ol, (4E)-, acetate	1016	1013	−	−	+	+	−	−	−	−	−
12	13.183	Butanoic acid, 1-methylbutyl ester	1022	1019	−	+	−	−	+	−	−	+	+
13	14.442	Butanoic acid, 3-methyl-,butylester	1043	1044	+	+	+	−	+	−	+	+	+
14	15.18	Butanoic acid, pentyl ester	1056	1055	+	+	+	+	+	−	+	−	−
15	17.592	Butanoic acid, 2-pentenyl ester,(Z)-	1098	1091	−	−	+	−	+	−	−	−	−
16	18.072	Pentanoic acid, 3-methylbutyl ester	1105	1104	+	+	+	−	+	−	+	−	−
17	19.39	Hexanoic acid, 3-hydroxy-, ethyl ester	1126	1124	−	−	+	+	+	−	−	−	−
18	21.037	Pentanoic acid, pentyl ester	1199	1183	−	+	+	−	+	−	−	−	−
19	23.494	Hexanoic acid, butyl ester	1190	1183	−	−	+	+	+	−	−	−	−
20	23.524	Butanoic acid, hexyl ester	1190	1183	−	+	−	−	−	−	−	−	+
21	23.8	2-Butenoic acid, hexyl ester	1194	1191	+	−	−	−	−	−	−	−	−
22	24.1	Butanoic acid, 4-hexen-1-yl ester	1199	1191	−	+	+	−	+	−	−	−	−
23	24.973	Isopentyl hexanoate	1212	1218	−	+	+	−	+	−	−	−	−
24	26.476	Benzene, 1,4-dimethoxy-2-methyl	1235	1229	+	−	−	−	−	−	−	−	−
25	26.895	Hexyl n-valerate	1242	1235	−	+	+	−	−	−	−	+	+
26	27.309	Isopentyl hexanoate	1248	1253	+	+	+	+	+	+	+	+	+
27	33.291	2(3H)-Furanone, dihydro-5-pentyl-	1341	1341	−	+	−	−	−	−	−	−	−
28	36.05	5-Dodecen-1-ol, acetate, (z)-	1386	1389	−	−	−	−	−	−	−	+	+
29	36.58	Decanoic acid, ethyl ester	1394	1393	+	−	−	−	−	−	−	−	−
30	58.383	Phthalic acid, isobutyl 2-pentyl ester	2069	2008	+	+	+	+	+	+	+	+	+
**2. Aromatic aldehyde**					1	2	1	1	0	1	0	0	0
1	5.913	2-Hexenal	846	838	+	+	+	+	−	+	−	−	−
2	26.334	Propanal, 3-cyclohexylidene-2-methyl-	1233	1227	−	+	−	−	−	−	−	−	−
**3. Alcohols**					4	3	1	5	2	3	5	0	0
1	18.018	2-Nonen-1-ol	1102	1105	−	−	−	+	−	+	−	−	−
2	24.501	3-Decan-1-ol, (E)-	1205	1233	−	−	−	+	−	−	+	−	−
3	31.8	1-Decanol, 2-ethyl-	1317	1350	+	−	−	−	−	−	−	−	−
4	36.833	1-Octanol, 2-butyl-	1398	1393	−	+	−	−	+	−	+	−	−
5	42.895	1-Decanol, 2-hexyl-	1496	1504	+	+	+	+	−	+	+	−	−
6	44.713	2-Hexyl-1-octanol	1590	1591	+	−	−	+	−	−	+	−	−
7	53.536	1-Hexadecanol, 2-methyl	1899	1890	+	+	−	+	+	+	+	−	−
**4. Hydrocarbons**					1	1	1	0	0	0	2	0	0
1	4.165	1,3,5-Cycloheptatriene	765	771	−	−	−	−	−	−	+	−	−
2	31.769	Decane,2,4,6-trimethyl-	1316	1318	−	+	−	−	−	−	+	−	−
3	33.325	Tridecane, 5-methyl-	1342	1349	+	−	+	−	−	−	−	−	−
**5. Ketones**					2	1	0	2	1	0	2	0	0
1	38.733	2-Butanone, 4-(2,6,6-trimethyl-1-cyclohexen-1-yl)-	1429	1427.8	+	−	−	+	+	−	+	−	−
2	39.722	5,9-Undecadien-2-one, 6,10-dimethyl-,(E)-	1445	1453	+	+	−	+	−	−	+	−	−
**6. Ethers**					2	1	1	1	1	2	1	1	1
1	26.476	Benzene, 1,4-dimethoxy-2-methyl	1235	1229	+	−	−	−	−	−	−	−	−
2	36.929	Benzene, 1,2-dimethoxy-4-(2-propenyl)-	1400	1402	−	−	−	−	−	+	−	−	−
3	45.776	Benzene, 1,2,4-trimethoxy-5-(1-propenyl)-,(Z)-	1622	1640	+	+	+	+	+	+	+	+	+
**7. Carboxylic acids**					0	0	0	0	1	0	0	0	0
1	33.442	2,2-Dimethylglutaric acid	1344	1345	−	−	−	−	+	−	−	−	−
**8. Diverse fun. Group**					1	3	1	0	1	1	0	2	1
1	30.263	2H-Pyran-2,3-diol, tetrahydro-, diacetate, cis-	1293	1364	−	+	+	−	−	−	−	−	−
2	33.783	Eugenol	1349	1337	−	−	−	−	−	−	−	+	+
3	35.877	Carbonic acid, dipentyl ester	1383	1358	−	+	−	−	−	−	−		
4	38.389	Chloroacetic acid, 2-ethylcyclohexyl ester	1423	1433								+	−
5	51.206	2-Propenoic acid, 3-(3,4-dimethoxyphenyl)-,(E)-	1774	1735	+	+	−	−	+	+	−		
**9. Chlorine-containing**					0	0	1	1	0	0	0	0	0
1	56.803	Octadecane, 1-chloro-	2037	2036	−	−	+	+	−	−	+		
Total					23	29	23	20	23	9	16	10	9

## Discussion

Genetic diversity and the development of genotype-specific markers are crucial for successful breeding and crop improvement. Banana breeding is a time-consuming and laborious process considering the requirements of time for crop cultivation, field size, and human resources. Therefore, the inclusion of marker-assisted breeding (MAB) can speed up the process of germplasm improvement and make it appealing to develop better performing commercial varieties^4,38^. Further, it can reduce the number of years consummated from pollination to pre-assessment of first fruit setting and yield, assessing several pollination cycles prior to screening for other biotic and abiotic stresses. Another advantage of MAB is the reduced cost of genetic screening and the wide range of sensing technology that can easily complement phenotyping^12^.

Chronically several limitations are faced by banana and other horticultural crop breeders as well as farmers from the tropics or subtropics, for example, with the widespread occurrence of misnomers^16,17^. However, misnomers are frequently grown as commercially cultivars, mislabelled with different names in local languages. To avoid such mislabeling and inappropriate branding, the characterization of misnomers (or synonyms) using molecular markers is essentially required. Thus a quick, simple, reproducible, and cost-effective method for cultivar identification and validation is required, which can be further developed as a certification method for cultured tissue plantlets of bananas. Moreover, it could also help in germplasm preservation and documentation of novel genetic resources^39^. Therefore, molecular markers hold promise and might help to encourage banana cultivation and smooth trade of authentic genetic material among banana-growing regions like the Marathwada region of Maharashtra, India. The present study offers unique information on molecular markers and volatile profiles for commercially grown cultivars from the Marathwada region, a significant banana cultivation belt within India. This particular region has a growing concern with several misnomers. Hence, a desired molecular marker, a short unique genomic DNA sequence or fragment that carries a conserved inter-specific motif, would be beneficial for appropriate varietal identification. Moreover, such DNA markers possess minimum intra-specific separation to enable the classification of several cultivars and species^40^; this approach is exploited in different species and cultivars of horticultural crops, including grapes^16,17^, pineapple^41^, and figs^42^. Our results have demonstrated that ISSR primer UBC-858 was found to be unique to Grand Nain, whereas the SSR maker CNMPF-13 was able to confirm the specific loci from the Ardhapuri cultivar, which could be further developed as biomarkers for DNA barcoding the plantlets produced through tissue culture for use in commercial cultivation or breeding programs. Since these DNA markers possessed strong discriminatory power, it could be helpful in the validation of uniformity among the plant population. The study showed that all the phenotypic characteristics in cultivated *Musa* sp. are geographical location and climate-specific. An earlier analysis of 68 banana accessions performed using RFLP of ITS fragments (digested with RsaI), produced steady, reliable, and distinctive patterns of polymorphic DNA in *Musa* sp. cultivars^43,44^. Likewise, ITS1–5.8S-ITS2 sequences^45^ have also been successfully exploited in molecular diversity analysis of *Musaceae*.

The aesthetic value of fruits is primarily defined by the constituent volatiles (volatilome), which contribute to the unique aroma and flavor. Moreover, the aroma or flavor of fruits generates significant appeal driving the urge for consumer acceptance, consumption and usage^25^. The distinctive chemical nature of aroma and flavor are formed by combination of several volatile small molecules. In the case of banana, its agreeable and classic aromatic smell has been studied relatively recently, and there have been over 150 volatiles found to bequeath a unique aroma^32^.

In the present study, we report over 33% of the volatiles from fruits of nine banana cultivars. This volatiles belonged to several classes of small molecules like esters, ketones, terpenes, and aldehydes. Previously, isoamyl and isobutyl esters and ketones have been reported to be dominant in banana^26^. In our study, we observed that over 56% contribution to banana flavor is contributed by esters and in cultivar, Grand Nain esters contributed to 78% of the volatiles. Similarly, cultivars namely Ardhapuri, Karpurvalli, Ankur, and Borkee Baista represented significant portion of volatiles belonging to ester compounds. Suggesting dominance of the ester molecules in contribution towards characteristics aroma in these cultivars. Additionally, alcohols and carboxylic compounds were also significant constituents of banana aroma. The SPME method used to extract volatiles is simple, quick, robust, and solvent-free^24^. The method was efficient for the recovery of volatiles and could be optimized quickly for any fruit volatilome analysis^26,32,46^. Since some of the volatile compounds are reported to impart resistance against plant pathogens^47^, additional research is warranted on extensive spatial and temporal volatilome analysis from banana crops to establish the underlying mechanism of volatile biosynthesis and genetic control of resistance against certain pathogens^47^. In the future, novel technologies such as CRISPR-Cas^47,48^ and nanotechnological applications^49–52^ could help to enhance banana crop improvement.

## Conclusions

Since banana is a polyploid and vegetatively propagated horticultural plant, genetic diversity and the development of genotype-specific markers are crucial for successful breeding and crop cultivation. The present study revealed a high polymorphism across the 20 banana genotypes evaluated with 11 RAPD, 11 ISSR, and 12 SSR primers. Phylogenetic and PCA clustered 20 banana genotypes into two significant clusters at 62% similarity level and 34% cumulative variation. AMOVA distributed 23% variation among the populations and 77% within the sub-populations with a significant FST value of 0.23, representing a high level of genetic differentiation. Interestingly, ISSR marker UBC-858 and SSR primer CNMPF-13 yielded a unique fingerprint for Grand Naine and Ardhapuri cultivars, respectively, and could be utilized as cultivar-specific biomarkers for clonal fidelity testing in the tissue culture industry. The volatile profiling revealed the presence of several volatiles in banana cultivars and highlighted the role of ester compounds in the characteristic aroma. Thus, the food processing industry could exploit the contribution of ester-like compounds in banana flavor.

## Supplementary Information

Supplementary Information.

## Data Availability

The accessions analyzed during the current study are available in the ICAR-NRC Banana repository (https://data.gov.in/resources/icar-publication-repository-date-nrc-banana-collection). The data generated and analyzed are presented in the figures or table of this submission.

## References

[1] D’Hont A (2012). The banana (Musa acuminata) genome and the evolution of monocotyledonous plants. Nature.

[2] Knoema. Bananas production quantity. https://knoema.com/atlas/topics/Agriculture/Crops-Production-Quantity-tonnes/Bananas-production (2022).

[3] Ssebuliba R (2006). Reproductive efficiency and breeding potential of East African highland (Musa AAA-EA) bananas. Field Crop. Res..

[4] Hippolyte I (2012). Foundation characteristics of edible Musa triploids revealed from allelic distribution of SSR markers. Ann. Bot..

[5] Mahadev SR, Kathithachalam A, Marimuthu M (2011). Efficient protocol for large-scale plantlet production from male floral meristems of *Musa* spp. cultivars Virupakshi and Sirumalai. In vitro Cell Dev. Biol. Plant.

[6] Čížková J (2013). Molecular analysis and genomic organization of major DNA satellites in banana (*Musa* spp.). PLoS ONE.

[7] Sardos J (2016). DArT whole genome profiling provides insights on the evolution and taxonomy of edible Banana (*Musa* spp.). Ann. Bot..

[8] Lorenzen J, Hearne S, Mbanjo G, Nyine M, Close T (2011). Use of molecular markers in banana and plantain improvement. Acta Hortic..

[9] Ruangsuttapha S (2007). Molecular phylogeny of banana cultivars from Thailand based on HAT-RAPD markers. Genet. Resour. Crop Evol..

[10] Nwakanma DC, Pillay M, Okoli BE, Tenkouano A (2003). PCR-RFLP of the ribosomal DNA internal transcribed spacers (ITS) provides markers for the A and B genomes in *Musa* L. Theor. Appl. Genet..

[11] Čížková J (2015). Molecular and cytogenetic characterization of wild Musa species. PLoS ONE.

[12] Perrier X (2011). Multidisciplinary perspectives on banana (*Musa* spp.) domestication. Proc. Natl. Acad. Sci..

[13] Christelová P (2017). Molecular and cytological characterization of the global *Musa* germplasm collection provides insights into the treasure of banana diversity. Biodivers. Conserv..

[14] Bakry, F., Carreel, F., Jenny, C. & Horry, J.-P. Genetic improvement of banana BT—Breeding plantation tree crops: Tropical species. in (eds. Jain, S. M. & Priyadarshan, P. M.) 3–50 (Springer, New York, 2009). 10.1007/978-0-387-71201-7_1.

[15] Ude G, Pillay M, Ogundiwin E, Tenkouano A (2003). Genetic diversity in an African plantain core collection using AFLP and RAPD markers. Theor. Appl. Genet..

[16] Upadhyay A, Kadam US, Chacko PM, Aher L, Karibasappa G (2010). Microsatellite analysis to differentiate clones of Thompson seedless grapevine. Indian J. Hortic..

[17] Upadhyay A, Kadam US, Chacko P, Karibasappa G (2010). Microsatellite and RAPD analysis of grape (*Vitis* spp.) accessions and identification of duplicates/misnomers in germplasm collection. Indian J. Hortic..

[18] Drapal M (2019). Metabolite profiling characterises chemotypes of *Musa* diploids and triploids at juvenile and pre-flowering growth stages. Sci. Rep..

[19] Hřibová E (2011). The ITS1–5.8S-ITS2 sequence region in the musaceae: Structure, diversity and use in molecular phylogeny. PLoS ONE.

[20] Pino JA, Febles Y (2013). Odour-active compounds in banana fruit cv. Giant Cavendish. Food Chem..

[21] Liu A-Z, Kress WJ, Li D-Z (2010). Phylogenetic analyses of the banana family (Musaceae) based on nuclear ribosomal (ITS) and chloroplast (*trnL-F*) evidence. Taxon.

[22] Pino JA, Castro-Benítez M, Winterhalter P (2020). Analysis of volatile compounds in baby banana peel and pulp (musa Acuminata AA Simmonds Cv. Bocadillo) in relation to the hyperpigmentation phenomenon. Int. J. Fruit Sci..

[23] Deng G (2021). Transcriptome and metabolome profiling provide insights into molecular mechanism of pseudostem elongation in banana. BMC Plant Biol..

[24] Majcher M, Jeleń HH (2009). Comparison of suitability of SPME, SAFE and SDE methods for isolation of flavor compounds from extruded potato snacks. J. Food Compos. Anal..

[25] Thaiphanit S, Anprung P (2010). Physicochemical and flavor changes of fragrant banana (*Musa* acuminata aaa group “Gross Michel”) during ripening. J. Food Process. Preserv..

[26] Zhu X (2018). Comparative study of volatile compounds in the fruit of two banana cultivars at different ripening stages. Molecules.

[27] Gawel NJ, Jarret RL (1991). A modified CTAB DNA extraction procedure for *Musa andIpomoea*. Plant Mol. Biol. Rep..

[28] Nei M (1973). Analysis of gene diversity in subdivided populations. Proc. Natl. Acad. Sci..

[29] Sneath, P. H. A. & Sokal, R. R. *Numerical Taxonomy. The Principles and Practice of Numerical Classification.* (1973).

[30] Hubisz MJ, Falush D, Stephens M, Pritchard JK (2009). Inferring weak population structure with the assistance of sample group information. Mol. Ecol. Resour..

[31] Earl D, vonHoldt BM (2011). Structure harvester: a website and program for visualizing structure output and implementing the Evanno method. Conserv. Genet. Resour..

[32] Facundo HVDV, Garruti DS, Cordenunsi BR, Lajolo FM (2013). Isolation of volatiles compounds in banana by HS-SPME: optimization for the whole fruit and pulp. Int. J. Biosci. Biochem. Bioinform..

[33] Hickey LT (2019). Breeding crops to feed 10 billion. Nat. Biotechnol..

[34] Borborah K (2020). Comparative analysis of genetic diversity in some non-commercial cultivars of *Musa* L. from Assam, India, using morphometric and ISSR markers. Int. J. Fruit Sci..

[35] Mertens A (2021). Genetic diversity and structure of Musa balbisiana populations in Vietnam and its implications for the conservation of banana crop wild relatives. PLoS ONE.

[36] Esteras C (2018). Fruit flesh volatile and carotenoid profile analysis within the *Cucumis melo* L. species reveals unexploited variability for future genetic breeding. J. Sci. Food Agric..

[37] Beekwilder, J. *et al.* Functional characterization of enzymes forming volatile esters from strawberry and banana. *Plant Physiol.* **135**(4), 1865–1878. 10.1104/pp.104.042580 (2004). PMID: 15326278; PMCID: PMC520758. 10.1104/pp.104.042580 PMC520758 15326278

[38] Christelová P (2011). A platform for efficient genotyping in *Musa* using microsatellite markers. AoB Plants.

[39] Uma S, Sathiamoorthy S, D. S. *Banana Indian Genetic Resources and Catalogue*. (National Research Center for Banana (NRCB) India (2005).

[40] Němečková A (2018). Molecular and cytogenetic study of east african highland banana. Front. Plant Sci..

[41] Hidayat T, Abdullah FI, Kuppusamy C, Samad AA, Wagiran A (2012). Molecular identification of malaysian pineapple cultivar based on internal transcribed spacer region. APCBEE Proc..

[42] Castro C, Hernandez A, Alvarado L, Flores D (2015). DNA barcodes in fig cultivars (*Ficus carica* L.) using ITS regions of ribosomal DNA, the psbA-trnH spacer and the matK coding sequence. Am. J. Plant Sci..

[43] Javed MA, Chai MAK, Othman RY (2002). Morphological characterization of malaysian wild banana *Musa* acuminata. Biotropia (Bogor).

[44] Bhat KV, Jarret RL, Rana RS (1995). DNA profiling of banana and plantain cultivars using random amplified polymorphic DNA (RAPD) and restriction fragment length polymorphism (RFLP) markers. Electrophoresis.

[45] Hapsari L, Ayu Lestari D (2016). Fruit characteristic and nutrient values of four Indonesian banana cultivars (*Musa* spp.) at different genomic groups. Agrivita.

[46] Zhang H (2020). Characterization of volatile profiles and marker substances by HS-SPME/GC-MS during the concentration of coconut jam. Foods.

[47] Hinge VR, Chavhan RL, Kale SP, Suprasanna P, Kadam US (2021). Engineering resistance against viruses in field crops using CRISPRCas9. Curr. Genomics.

[48] Kadam US, Shelake RM, Chavhan RL, Suprasanna P (2018). Concerns regarding ‘off-target’ activity of genome editing endonucleases. Plant Physiol. Biochem..

[49] Kadam, U. S., Lossie, A. C., Schulz, B. & Irudayaraj, J. *Gene expression analysis using conventional and imaging methods*. *DNA and RNA Nanobiotechnologies in Medicine: Diagnosis and Treatment of Diseases*. 141–162 (Springer, Berlin, 2013). 10.1007/978-3-642-36853-0_6.

[50] Kadam US, Schulz B, Irudayaraj JMKK (2017). Multiplex single-cell quantification of rare RNA transcripts from protoplasts in a model plant system. Plant J..

[51] Kadam US, Chavhan RL, Schulz B, Irudayaraj J (2017). Single molecule Raman spectroscopic assay to detect transgene from GM plants. Anal. Biochem..

[52] Kadam U, Moeller CA, Irudayaraj J, Schulz B (2014). Effect of T-DNA insertions on mRNA transcript copy numbers upstream and downstream of the insertion site in arabidopsis thaliana explored by surface enhanced raman spectroscopy. Plant Biotechnol. J..

