# Supplementary material for: Assessment of genetic diversity and volatile content of commercially grown banana (Musa spp.) cultivars
Source: Sci Rep. 2022 May 13;12:7979. doi: 10.1038/s41598-022-11992-1 (PMC9106755; doi:10.1038/s41598-022-11992-1)
Supplement: Supplementary file 1 — Supplementary Information. [file 41598_2022_11992_MOESM1_ESM.pptx]

## Slide 1
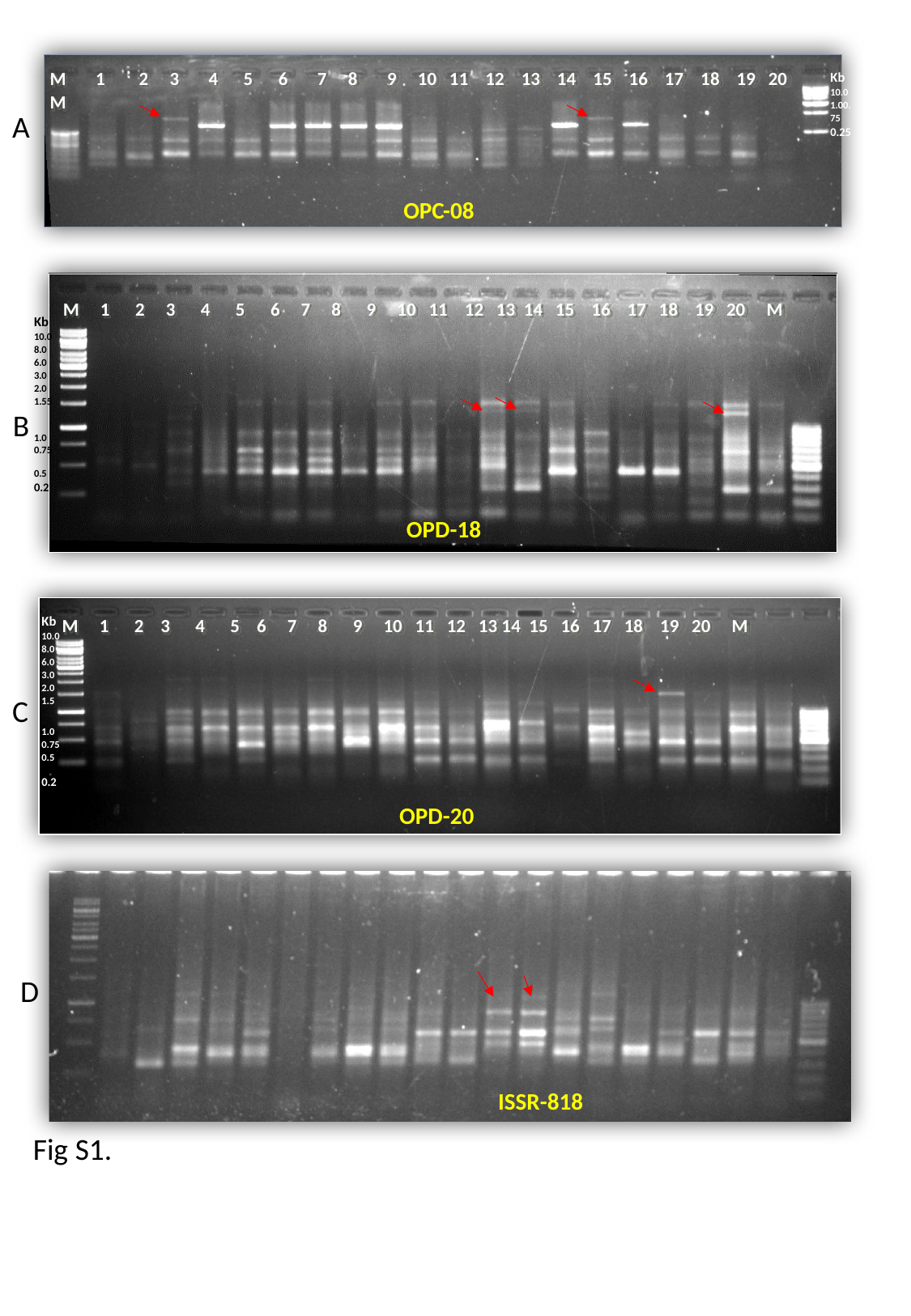

M 1 2 3 4 5 6 7 8 9 10 11 12 13 14 15 16 17 18 19 20 M
Kb 10.0 1.00.75 0.25
OPC-08
A
M 1 2 3 4 5 6 7 8 9 10 11 12 13 14 15 16 17 18 19 20 M
Kb 10.0 8.0 6.0 3.0 2.0
1.55
1.0
0.75
0.5
0.2
OPD-18
B
M 1 2 3 4 5 6 7 8 9 10 11 12 13 14 15 16 17 18 19 20 M
Kb 10.0 8.0 6.0 3.0 2.0
1.5
1.0
0.75
0.5
0.2
OPD-20
C
D
ISSR-818
ISSR-818
Fig S1.

## Slide 2
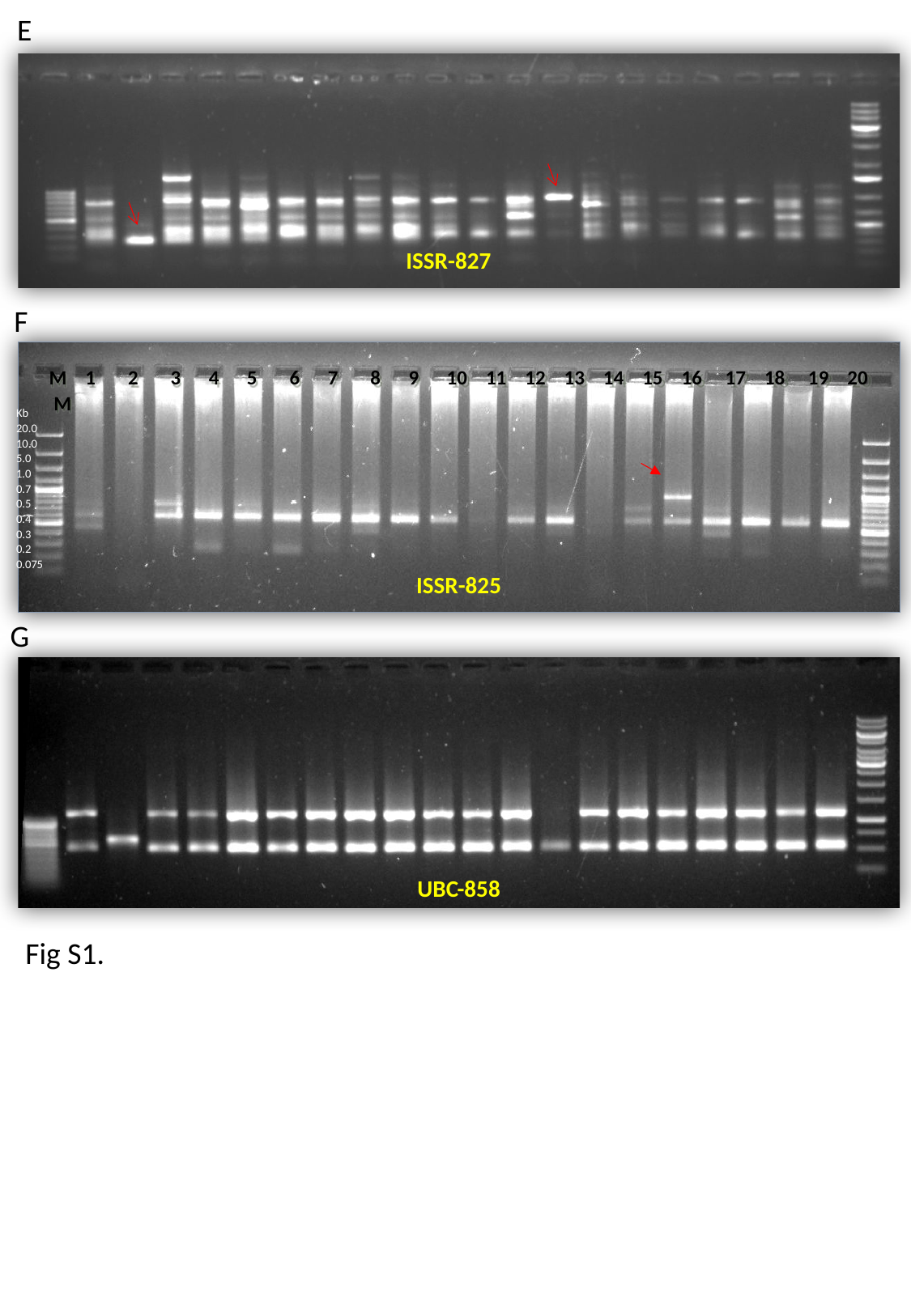

E
ISSR-827
F
M 1 2 3 4 5 6 7 8 9 10 11 12 13 14 15 16 17 18 19 20 M
Kb 20.0
10.0
5.0 1.0 0.7 0.5 0.4 0.3 0.2 0.075
ISSR-825
G
UBC-858
Fig S1.

## Slide 3
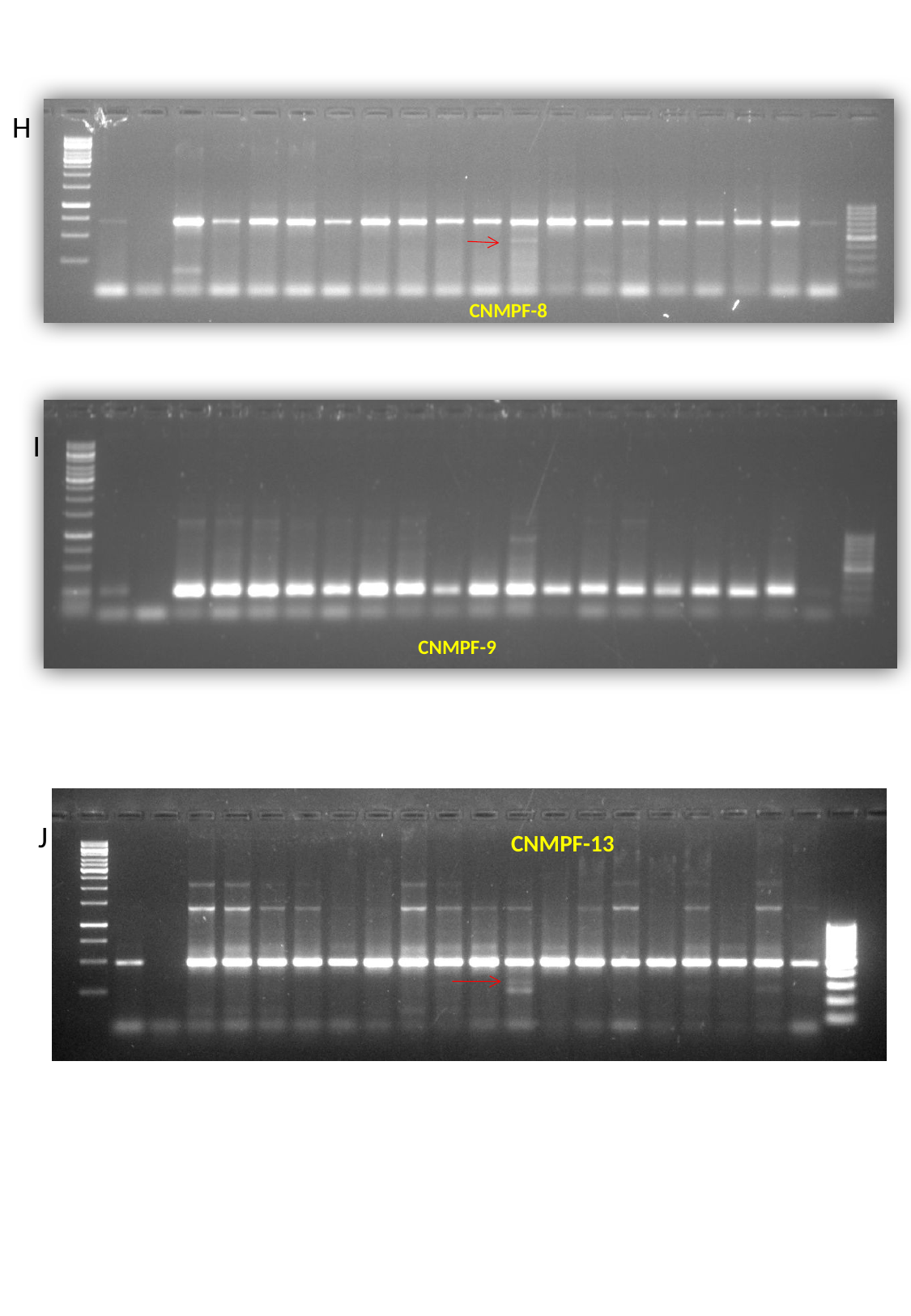

H
CNMPF-8
I
CNMPF-9
J
CNMPF-13

## Slide 4
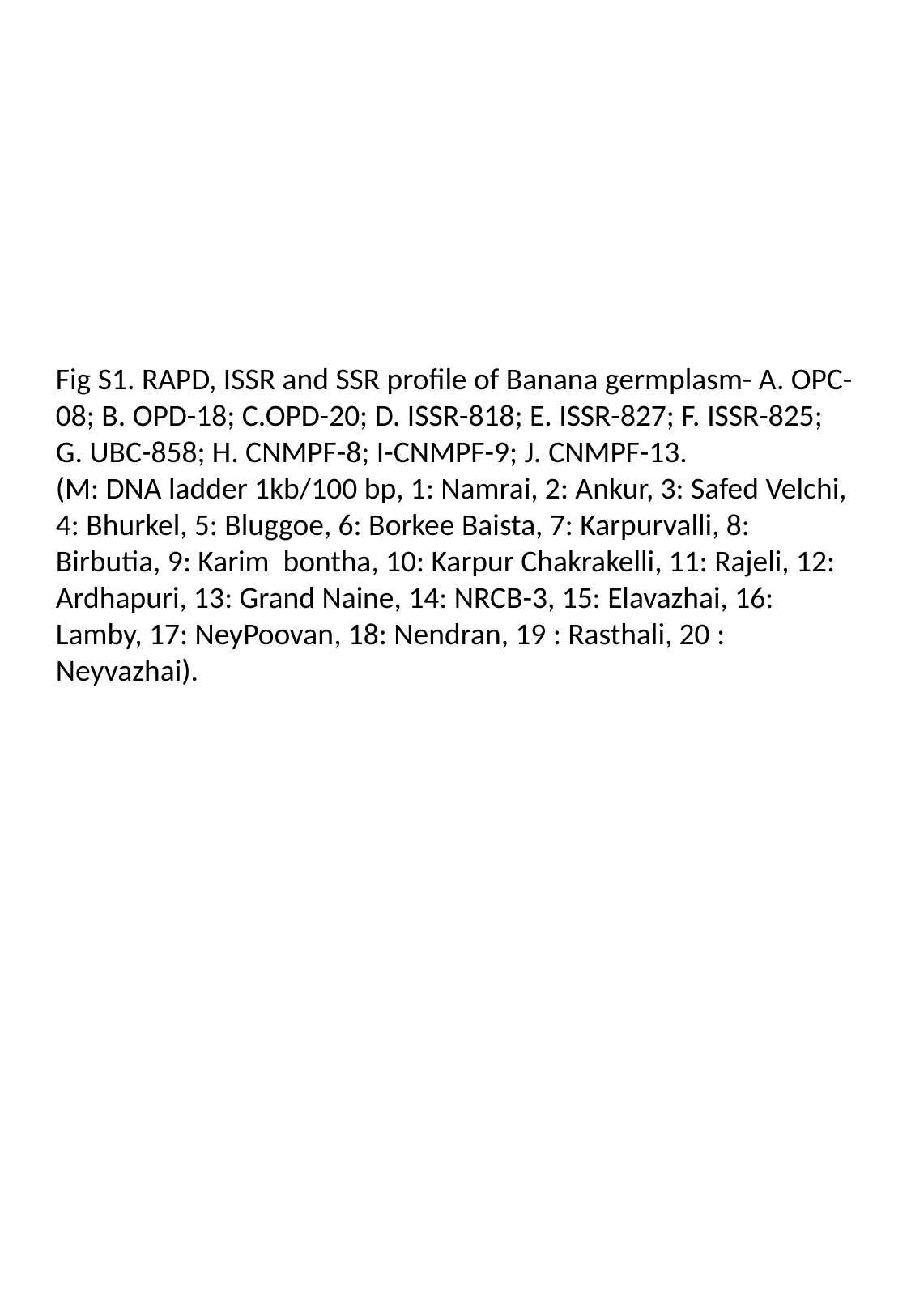

Fig S1. RAPD, ISSR and SSR profile of Banana germplasm- A. OPC-08; B. OPD-18; C.OPD-20; D. ISSR-818; E. ISSR-827; F. ISSR-825; G. UBC-858; H. CNMPF-8; I-CNMPF-9; J. CNMPF-13.
(M: DNA ladder 1kb/100 bp, 1: Namrai, 2: Ankur, 3: Safed Velchi, 4: Bhurkel, 5: Bluggoe, 6: Borkee Baista, 7: Karpurvalli, 8: Birbutia, 9: Karim bontha, 10: Karpur Chakrakelli, 11: Rajeli, 12: Ardhapuri, 13: Grand Naine, 14: NRCB-3, 15: Elavazhai, 16: Lamby, 17: NeyPoovan, 18: Nendran, 19 : Rasthali, 20 : Neyvazhai).
